# Applying the DSM-5 Criteria for Gambling Disorder to Online Gambling Account-Based Tracking Data: An Empirical Study Utilizing Cluster Analysis

**DOI:** 10.1007/s10899-021-10080-9

**Published:** 2021-10-11

**Authors:** Maris Catania, Mark D. Griffiths

**Affiliations:** 1 Kindred Group, Level 6, The Centre, Tigne Point, Sliema, TPO001 Malta; 2 International Gaming Research Unit, Psychology Department, Nottingham Trent University, 50 Shakespeare Street, Nottingham, NG1 4FQ UK

**Keywords:** Online gambling, Internet gambling, Responsible gambling, Behavioural tracking, Gambling disorder, DSM-5 criteria

## Abstract

The emergence of online gambling has raised concerns about potential gambling-related harm, and various measures have been implemented in order to minimise harm such as identifying and/or predicting potential markers of harm. The present study explored how the nine DSM-5 criteria for gambling disorder can be operationalised in terms of actual online gambling behaviour using account-based gambling tracking data. The authors were given access to an anonymised sample of 982 gamblers registered with an online gambling operator. The data collected for these gamblers consisted of their first three months’ gambling activity. The data points included customer service contacts, number of hours spent gambling, number of active days, deposit amounts and frequency, the number of times a responsible gambling tool (such as deposit limit) were removed by the gamblers themselves, number of cancelled withdrawals, number of third-party requests, number of registered credit cards, and frequency of requesting bonuses through customer service (i.e., the number of instances of ‘bonus begging’). Using these metrics, most of the DSM-5 criteria for gambling disorder can be operationalized (at least to some extent) using actual transaction data. These metrics were then applied to a sample of online gamblers, and through cluster analysis four types of online gambler based on these metrics (non-problem gamblers, at-risk gamblers, financially vulnerable gamblers, and emotionally vulnerable gamblers) were identified. The present study is the first to examine the application of the DSM-5 criteria of gambling disorder to actual gambling behaviour using online gambling transaction data and suggests ways that gambling operators could identify problem gamblers online without the need for self-report diagnostic screening instruments.

## Introduction

The emergence of online gambling has not only resulted in higher availability (Canale et al., 2016) but also concerns about potential gambling-related harm (Dragicevic et al., 2015). Various measures have been implemented in order to minimise harm, such as responsible gambling (RG) tools, policies, and protocols (Wood & Griffiths, 2010). RG tools can help gamblers reduce or control their gambling (Wood et al., 2014). These tools have become more available in recent years, as evidenced in a study by Smeaton and Griffiths (2004) which found that out of a sample of 30 operators, only one operator offered self-exclusion, whereas in a study 13 years later where 50 operators were examined, 43 operators offered self-exclusion (Bonello & Griffiths, 2017).

Online behavioural tracking can help in minimising harm by identifying and/or predicting potential problem gambling (Haeusler, 2016). Moreover, through the examination of behavioural tracking data, researchers and gambling operators may understand better how online gamblers behave and act over an extensive time period (Griffiths, 2014). Online gambling operators can use this information to identify and help online problem gamblers, either through an already established behavioural tracking tool such as *PlayScan* (Griffiths, 2009), or develop their own, such as *Kindred’s* Player Safety—Early Detection System (Kindred, 2019).

By using such tracking technology, limitations in diagnostic criteria may be overcome. These include overcoming weaknesses associated with self-reporting such as providing false information or answering questions in a socially desirable manner (Griffiths, 2009). In order to be able to examine and potentially predict online problem gambling, most research has focused upon on using voluntary self-exclusion as a proxy measure for problem gambling (e.g., Braverman & Shaffer, 2012; Haefeli, et al., 2011; Percy et al., 2016). However, this approach may not be ideal because gamblers may be using self-exclusion for various reasons other than problem gambling, such as frustration and annoyance with the gaming operator (Griffiths & Auer, 2016).

Gambling disorder is a psychiatric disorder which results in maladaptive patterns of gambling behaviour (Grant et al., 2017). In the most recent (fifth) edition of the Diagnostic and Statistical Manual of Mental Disorders (DSM-5), gambling disorder was identified as a behavioural addiction (American Psychiatric Association, 2013). In order to be classed as experiencing gambling disorder individuals have to endorse four of more of nine criteria which can be classed at a number of severity levels: four to five criteria result as mild, six to seven as moderate, and eight or nine as severe (American Psychiatric Association, 2013; Grant et al., 2017).

To the best of the authors’ knowledge, the application of the extent to which the DSM criteria for gambling disorder can be identified using online gambling tracking data has only ever been examined theoretically and the analysis was based on the DSM-IV criteria (i.e., Griffiths, 2012; Griffiths & Whitty, 2010). More specifically, the authors examined the extent to which the behaviours listed in the DSM-IV criteria for pathological gambling could be identified using actual online gambling behaviour (as opposed to the consequences of it). To date, the application of the DSM-5 criteria to actual online gambling has not been examined. Although it was previously argued that most of the DSM criteria cannot be applied to gambling tracking data (Griffiths, 2012; Griffiths & Whitty, 2010), the present study explores new online gambling indicators that may be applicable to the DSM-5 criteria for gambling disorder that were not considered in the previous theoretical analyses.

### DSM-5 Criteria for Gambling Disorder

In this section, each of the nine DSM-5 criteria is explored alongside an assessment of the extent to which such behaviour can be visible online using account-based tracking data. The examples of operationalizing each criterion into behavioural measures were developed by the authors including the first author who works in the online gambling industry, and through conversations with experts from the online gambling industry, regulatory bodies, reformed problem gamblers and other gambling researchers, as well as examples from the extant gambling literature.

1. *“Is often preoccupied with gambling (e.g., having persistent thoughts of reliving past gambling experiences, handicapping or planning the next venture, thinking of ways to get money with which to gamble)” (i.e., preoccupation):*
  This criterion refers to when the individual experiences persistent thoughts of past gambling experiences and devises ways on how to get money which can be used for gambling. Most importantly, preoccupation can be operationalized as the amount of time and money that an individual spends gambling (Griffiths, 2012). This can be easily monitored by a gambling operator simply by examining the number of hours that the gambler spends daily on their website, the number of days that have gambling activity over a specific time period, and the amount of money that is deposited daily into their account. As highlighted by Griffiths (2012), individuals that gamble for long periods of time continuously may be experiencing problematic gambling in an attempt to escape from other things in their lives. McCormack et al. (2013) also reported that problematic gambling was more likely among those who were gambling online for more than four hours in single sessions. According to Gainsbury et al. (2019), higher overall engagement with gambling is a key predictor of harms related to gambling. This is also evident in a study by Dragicevic et al. (2015) where those who eventually voluntarily excluded themselves from gambling at the website examined lost significantly more money gambling compared to the control group who did not exclude themselves. The amount of money deposited daily by gamblers has also been associated with problem gambling-related self-exclusion (Ukhov et al., 2021). When gamblers are preoccupied with gambling, they may also contact the gambling operator at a higher frequency because they are consistently thinking about their gambling account. Through efficient record keeping, this criterion can easily be recorded and monitored by the gambling operator. This higher frequency of communication is in accordance to the study conducted by Haefeli et al. (2011) who found that although gamblers might not directly communicate financial problems that they are experiencing, these issues become burdensome and would entail the gambler contacting the operator even more, and with more urgency.
2. *“Needs to gamble with increasing amounts of money in order to achieve the desired excitement” (i.e., tolerance):*
  This criterion refers to the individual needing to gamble with increasing amounts of money to achieve the desired mood state (such as arousal or escape). According to Griffiths (2012), this can be seen when online problem gamblers change their behaviour by increasing the amount of time or money spent gambling over time. It is evident that this behaviour can be easily tracked online especially if an operator examines the number of days when the gambler is playing, for example a gambler goes from playing once weekly to three times weekly to most days weekly over a number of months. A gambling operator could also monitor the amount of money deposited daily or the amount of money lost daily. If this is increasing, it may be a sign of tolerance among gamblers.
3. *“Is restless or irritable when attempting to cut down or stop gambling” (i.e., withdrawal):*
  This criterion refers to when the individual becomes highly restless, moody and/or irritable when there is an attempt to cut down or refrain from gambling. Griffiths (2012) highlights that this might be difficult to spot online, apart from looking at aggressive communication the gambler might engage in within online gambling chatrooms. This is also evident in a study by Haefeli et al. (2011) who reported that gamblers who eventually self-excluded, showed abusive communication with the gambling operator’s staff members. Therefore, some indicators of withdrawal symptoms may be observed indirectly by the gambling operator.
4. *“Has made repeated unsuccessful efforts to control, cut back, or stop gambling” (i.e., loss of control):*
  This criterion refers to when the individual has made a number of efforts which were unsuccessful to control, limit, or stop the gambling. Gamblers may try to protect themselves by withdrawing their winnings or the balance on the gambling account, in order not to spend all their money. Although this appears to be an attempt to control their gambling, if the withdrawal is cancelled in order to re-invest the money in gambling, such behaviour may be a potential indicator of loss of control. Another possible indicator of loss of control may be gamblers who suddenly stop using responsible gambling tools such as limit-setting. Gamblers have the possibility of setting up RG tools such as limits to help them control and limit their gambling. Most operators have the possibility for the gamblers to set up daily, weekly and/or monthly limits (Bonello & Griffiths, 2017). In the same manner that a gambler may set up RG limits, they may in most cases, easily remove it online, albeit having a cooling-off period for the cancellation to be active. This may be a possible indicator of loss of control since gamblers have tried to control and limit their gambling but may be removing such options in order to have no restrictions to their gambling.
5. *“Often gambles when feeling distressed (e.g., helpless, guilty, anxious, depressed) (i.e., escapism):*
  This criterion refers to when individuals use gambling as a way of relieving themselves from a psychological mood state which can range from depression to everyday stress. This is very difficult to spot online unless gamblers make such admissions to customer services staff or in online gambling chatrooms that are built into some online games such as online bingo and online poker.
6. *“After losing money gambling, often returns another day to get even (“chasing” one’s losses) (i.e., chasing):*
  This criterion refers to when individuals gamble with increasing amounts of money to get even and to regain they money they have lost. This is a key indicator of potential problem gambling which can be easily spotted online, through an increase in gambling activity and monetary depositing. This can be done repeatedly and due to the transactional nature of the online data available, it is relatively easy to spot. In most cases, such activity can be examined on a daily basis or during in-session gambling.
7. *“Lies to conceal the extent of involvement with gambling” (i.e., lying):*
  This criterion refers to when individuals lie to hide the real involvement they have with gambling. This is difficult to spot online as it would be reliant on the communication with the gambler. The only possibility to identify if gamblers are lying is if they provide information that does not match their activity on the gambling account. Such an example would be if gamblers communicate with customer services that they did not deposit any money in their gambling account the day before, whereas the transactional data show they clearly did. Another example is where gamblers lie about their account being hacked and that the money lost on the account was not theirs.
8. *“Has jeopardised or lost a significant relationship, job, or educational or career opportunity because of gambling” (i.e., risking significant relationships and occupational/educational opportunities):*
  This criterion refers to where individuals compromise their relationships as a result of their gambling and/or their gambling compromises occupational and/or educational opportunities. This is very difficult for an online gambling operator to spot online. The only way an operator may be able to confirm such behaviour would be through a ‘third party request’. This is when a third party contacts the gambling operator to get information about and/or exclude a customer they have a relationship with. In most jurisdictions, the gambling operator cannot confirm any information to the third party due to data protection issues, but this incident would be still be recorded by the gambling operator. In some jurisdictions (e.g., Singapore), it is possible for a gambler to be excluded through the third party’s request, but in most jurisdictions, this is not possible.
9. “*Relies on others to provide money to relieve desperate financial situations caused by gambling” (i.e., bailout):*
  This criterion refers to when the individual relies on others to relieve them from desperate financial situations. In these cases, it is difficult to know if a gambler received money from someone else unless there is a direct third-party deposit on the gambler’s account. Bailout may also be visible through a higher number of credit cards on the account. Through credit cards, gamblers can borrow funds which can be used to gamble thinking that if they win, they may be able to relieve themselves from the difficult financial situation they might have placed themselves in. In other instances, gamblers may have run out of money and in order to try and win the money back to relieve their financial situation, they ask for a bonus. A bonus refers to money or credit given by the gambling operator for gamblers to use on their account. This is known within the industry as ‘bonus begging’. When a gambler requests a bonus, it does not necessarily mean that the operator granted the bonus but ‘bonus begging’ may be viewed conceptually as a bail-out given the definition provided in the DSM-5 for gambling disorder.

### The Present Study

The aim of the present study was to examine the aforementioned proposed behavioural measure indicators and identify their occurrence across an anonymised sample from an online gambling operator.

## Methods

### Participants and Procedure

The participants in the present study were all the UK customers (N = 982) who registered with *Unibet* between September 1, 2017, until December 31, 2017. For each of these accounts, the account history of the first three months from their registration date was obtained*.* In order to focus on more regularly active accounts, the only inclusion criterion was that the gamblers had to have played for at least three weeks in the first three months on their gambling account. The data comprised gambling activity from September 2017 to March 2018. This group of customers comprised 86.8% males (n = 852) and 13.2% females (n = 130). The majority of the gamblers were aged 26–35 years (n = 396; 40.33%), followed by those aged 18–25 years (n = 259, 26.38%), and those aged 36–45 years (n = 242; 24.64%). The age group with least number of gamblers was of those aged 66 years or older (n = 24; 2.44%) followed by those aged 56–65 years (n = 61, 6.21%).

### Gambling Website Description and Procedure

The present authors were given access to an anonymised dataset of customers at *Unibet* in order to carry out secondary analysis*.* This online gambling company offers a variety of online products including poker, casino games, and sports betting. The data collected for these gamblers consisted of their first three months’ gambling activity. The data points included customer service contacts, number of hours spent gambling, number of active days, deposit amounts and frequency, the number of times a responsible gambling tool (such as deposit limit) were removed by the gamblers themselves, number of cancelled withdrawals, number of third-party requests, number of registered credit cards, and frequency of requesting bonuses through customer service (i.e., the number of instances of ‘bonus begging’). The study was given ethical approval by the research team’s university ethics committee. Analysis of all data was carried out using SPSS Version 27. The data were all anonymised so that no customer profiles were identifiable to the researchers.

### Data Analysis

The behavioural data measures noted in the aforementioned section were identified for all the gamblers in the anonymised sample. For each behavioural measure, the data were extracted, and descriptive statistics are presented below. In order to ensure comparability, these measures were applied to a z-normalisation. The identification of these groups was based on two-step cluster analysis, which helped to identify natural groupings within a large dataset. The two-step clustering system represents an algorithm which is scalable and allows large datasets to be handled. Two-step clustering was used which measures distance based on the likelihood of participants being combined together (Melia & Heckerman, 1998). This approach is advantageous as it reduces the distance between all potential matches, but disadvantageous in that it does not consider the number of cases (Conry et al., 2011). The clusters were named in an objective manner based on the findings per cluster. For each DSM-5 criterion, descriptive statistics on each criterion are presented.

## Results

*Preoccupation* This criterion was examined in four different ways*.* The first way that preoccupation was assessed was the number of hours that the gamblers spent on their gambling account. In the span of three months, the average number of hours that a gambler spent online was 126 h, which would correspond to just above five days. Therefore, on average customers spent five days online out of three months. This ranged from 11 h to one gambler spending 853 h online (equivalent to almost 36 days). The second way preoccupation was assessed was the total number of days during this period when the individuals were active on their gambling account. On average, gamblers were active for 50 days out of the three-month period, which corresponds to slightly more than half the number of days of the days sampled. The fewest number of active gambling days was 21 days, but this was due to the fact that one of the inclusion criteria to be included in the study was having at least three weeks’ activity. The highest number of days was one gambler who accessed his account every day. The third way of assessing preoccupation was examining the amount of money deposited into the account. When looking at the depositing days only, on average £142 per gambler was deposited daily. The minimum daily deposit was £5 and the maximum was £15,499.06.

Finally, gamblers’ communication with customer services (CS) was examined. For each gambler a unique identifier was given, and this was matched with their communication with CS. For this study, only live chat correspondence was analysed. For each gambler, the number of CS contacts was recorded during the period analysed. On average, customer service was contacted 1.4 times per gambler, but this varied significantly. Most gamblers did not contact customer service at all (N = 658; 67%), whereas there were a number of gamblers who contacted customer service more than 20 times in the space of three months (N = 12, 1.2%), with one gambler contacting customer service 82 times.

*Tolerance* For this criterion, two potential indicators were assessed: increase in the number of active days over time and the increase in the number of monetary deposits over time. For each criterion, the totals were calculated on a weekly basis, and then the increase in the totals were calculated. When comparing the total number of days where the gambler was active on the gambling website during the first week of activity, compared to the total number of days where the gambler was active on the gambling website during the last week of activity. Over four-fifths of the sample (82.28%) increased their number of active playing days from one day in the first week of the study period to six active days in the final week of the study period (i.e., a six-fold increase for most players over time). For a much smaller group of gamblers, this did not increase at all and the number of active days stayed constant week-on-week throughout the three-month period. When observing the increase in the number of monetary deposits done on a weekly basis, on average, the number of monetary deposits per week was also observed. When comparing the total number of deposits made in the first week to the total number of deposits made in the last week, the number of deposits either did not increase at all or ranged from one to seven (for example, the score would be an increase by seven if a gambler deposited three times in the first week, in the last week there were 21 deposits). Therefore, some gamblers had a constant number of deposits week-on-week, whereas some gamblers increased the number of deposits sevenfold by the end of the study period. On average, the number of deposits increased fourfold.

*Withdrawal Symptoms* Given that withdrawal symptoms cannot directly be assessed using account-based data*,* the authors examined communication incidents where the gambler was abusive with one of the customer service representatives (i.e., making the assumption that frustration, moodiness and/or irritability might be indicative of withdrawal symptoms). Any communication which included personal attack on the customer service agent, was of a threatening nature, or was an insulting communication was noted. Most gamblers did not engage in this type of communication apart from 11 gamblers, and the incidents ranged from one to 13 incidents. Out of these 11 gamblers, five gamblers displayed abusive communication once. The highest recorded number of abusive communication incidents was nine by one gambler and 13 by another.

*Loss of Control* The loss of control criterion was operationalised as a gambler deciding to stop using a responsible gambling tool that they had voluntarily set up. These are cases where gamblers had controlled their gambling by activating an RG tool but then later deciding to remove it. Such an example would include a gambler who chose to set a daily wagering limit of £10 and then removing this limit so that they can deposit more than originally planned (albeit after a cooling-off period). Only nine gamblers removed an RG tool twice or more over the study period. Another measure which was considered for loss of control was cancelled withdrawals. The reason for including cancelled withdrawals is because this behaviour shows a lack of restraint and potential loss of control. Gamblers can request to withdraw funds (typically winnings) from their gambling account back into their personal financial account, and this process is typically quick (with gamblers receiving their money on the same day). However, gamblers may cancel this withdrawal request. Such funds are then returned back to their gambling account for the money to be used to gamble. The average number of cancelled withdrawals was 1.57 per gambler. Whereas the majority of gamblers did not cancel withdrawals, there were 39 gamblers (3.97%) that cancelled their withdrawals. The highest number of cancelled withdrawals among the gamblers were 65, 92, and 179 by three of the gamblers.

*Escapism* As aforementioned, escapism is difficult to detect unless the gambler discloses that gambling is being used as a form of escapism to other gamblers in a chatroom or to CS. Through the analysis of the communication of this sample of gamblers, no instance of escapism was mentioned by a gambler.

*Chasing* In order to examine chasing, each financial deposit per gambler was observed and the change in amount was recorded. In this case, for each gambler, the first initial monetary deposit was recorded. Then, the highest monetary deposit was recorded during the study period. These two values were then compared. On average, gamblers increased their initial deposit threefold (therefore, if a gambler’s first deposit was of £50, their highest deposit during the study period was £150), although almost 30% of the gamblers (n = 281) did not increase their initial deposit at all.

*Lying* Lying is one of the most difficult criteria to spot in online gambling, especially since this criterion is to conceal the extent of gambling from others. No data from any metric collected found any instance of lying.

*Risking Significant Relationships* In order examine this criterion, the researchers examined correspondence where a third-party contacted customer services in an attempt to close the individual’s gambling account. Although rather uncommon, and the gambling operator cannot do much due to data protection issues, the operator still recorded these instances. There were four third-party contacts for different cases (concerning a unique gambler every time), whereas in one case, two third-party individuals contacted customer services concerning the same gambler.

*Bailout* Since credit cards may result in gamblers getting credit to fund their gambling and potentially using this credit to continue gambling in an attempt to relieve their financial burden, this was used to assess bailout. When looking at the number of gamblers using credit cards, the majority of gamblers (n = 851; 86.7%) had used a credit card at some point during the period examined. The majority of the gamblers (n = 616; 62.7%) had only registered one credit card on their account. The highest number of registered credit cards was eight which was for one gambler only. Requesting a bonus was also examined as bailout because money/credit is being asked for by the gambler to a third party (in this case, the gambling operator). In these instances, the gambler asks for free money in order to gamble with it online, probably due to being a desperate state of finding ways of funding their gambling but also to potentially win back some of the money that they have lost gambling. At the time of the study, requests by gamblers for a bonus were usually declined by the operator, especially if the requests were repetitive because such actions are flagged as a potential indicator of problem gambling by the operator. With regards to bonus begging, this was recorded by examining every customer correspondence and recording the number of times a gambler requested a bonus. The average number of requests for bonuses was 0.5 per gambler but this was heavily skewed. The maximum number of requests for a bonus was 62 times. Compared to the whole population, only 12 gamblers requested bonuses more than 10 times, with the highest number of requests being 35, 46, and 62 by three gamblers.

## Cluster Profiles

The data were analysed by using SPSS (Version 27.0). The clusters were identified amongst 982 participants using the SPSS two-step clustering algorithm. This algorithm is best used for large amounts of data, and it identifies which combinations are most logical. In the initial step of the cluster analysis, the data are sorted into pre-clusters. These can be found in Table 1. The SPSS the algorithm examines the cases based on the distance measures between the z-scores to determine whether a new cluster should be formed or if the case should be included in an already existing cluster. For each gambler, each aforementioned behavioural variable mentioned was calculated, and in order to ensure that the values could be compared, these were converted to z-scores. Four clusters emerged, with samples sizes of 646 participants (65.78%; non-problem gamblers), three participants (0.31%; financially vulnerable gamblers), nine participants (0.92%; emotionally vulnerable gamblers), and 324 participants (32.99%; at-risk gamblers). Part of the two-step cluster analysis, Table 2 shows the final clusters formed. The findings show is that there were four distinct clusters. Cluster 1 contained two-thirds of the gamblers in the present sample. All the gamblers in Cluster 1 had lower mean values on all the metrics compared to all the other gamblers in the present sample (i.e., they were gambling at much safer and non-problematic levels compared to the other three groups). The remaining three other clusters comprised gamblers who had elevated scores on some or most of the gambling metrics. The specific differences are discussed below. The figures in Tables 1 and 2 both show z-scores.

**Table 1:** Initial clusters formed based on the gambling metrics assessed

	Cluster 1	Cluster 2	Cluster 3	Cluster 4
Communication with customer services	− 0.293	− 0.087	− 0.087	− 0.293
Hours spent gambling	− 0.589	0.198	1.147	0.035
Number of active days gambling	− 0.208	1.058	0.080	− 0.151
Daily deposit amount	− 0.073	24.379	0.001	− 0.193
Increase in number of days gambling over time	0.500	0.500	0.500	0.500
Increase in number of deposits over time	− 0.952	0.015	1.467	0.015
Removal of RG tools	− 0.248	− 0.248	2.650	− 0.248
Cancelled withdrawals	22.130	− 0.068	− 0.068	− 0.068
Abusive communication with customer services	− 0.072	− 0.072	24.067	− 0.072
Chasing losses	− 0.077	23.846	0.103	− 0.137
Third-party calls	− 0.068	− 0.068	− 0.068	22.130
Number of registered credit cards	− 0.228	0.963	3.343	− 0.228
Frequency of bonus begging	− 0.151	− 0.151	6.720	0.148

**Table 2:** Final clusters formed based on the gambling metrics assessed

	Cluster 1	Cluster 2	Cluster 3	Cluster 4
Communication with customer services	− 0.141	0.050	− 0.110	0.283
Hours spent gambling	− 0.455	2.260	0.178	0.881
Number of active days gambling	− 0.479	1.077	0.291	0.937
Daily deposit amount	− 0.058	13.801	− 0.151	− 0.009
Increase in number of days gambling over time	− 0.231	0.500	− 0.109	0.458
Increase in number of deposits over time	− 0.341	0.661	0.338	0.665
Removal of RG tools	− 0.082	− 0.248	0.074	0.164
Cancelled withdrawals	− 0.016	− 0.068	− 0.068	0.035
Abusive communication with customer services	− 0.069	− 0.072	5.705	− 0.020
Chasing losses	− 0.060	14.803	− 0.106	− 0.014
Third-party calls	− 0.033	− 0.068	− 0.068	0.070
Number of registered credit cards	− 0.051	1.360	0.830	0.066
Frequency of bonus begging	− 0.081	− 0.151	8.080	− 0.061

## Cluster 1—Non-problem Gamblers

**Fig. 1:**
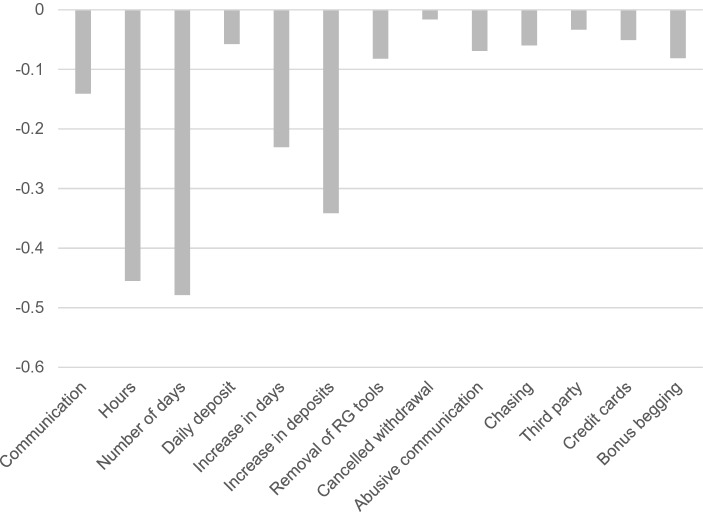
Behavioural metrics of the non-problem gambler cluster

The non-problem gambling cluster (n = 646, 65.78%) comprised the majority of the gamblers examined in the present study. The scores where all converted to z-scores, where negative scores refer to values that are below the mean for the whole group. When looking at each DSM-5 criterion, gamblers in this group scored negatively on all the criteria. More specifically, compared to all the other gamblers in the present sample, the gamblers in this cluster had lower mean scores on all the gambling metrics. Each of the following tables shows the z-scores along the y-axis with each DSM-5 criterion for gambling disorder along the x-axis (Fig. 1).

## Cluster 2—Financially Vulnerable Gamblers

**Fig. 2:**
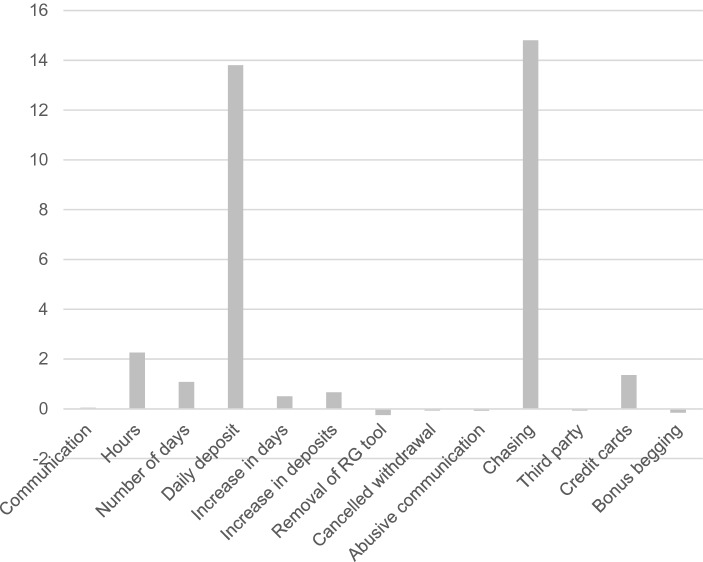
Behavioural metrics of the financially vulnerable gambler cluster

The financially vulnerable cluster comprised only three gamblers (0.31%) and this consisted of gamblers who predominantly displayed higher values on the criteria related to gambling expenditure such as the number of daily money deposits and the increase in daily money deposits over time on the chasing criteria. The number of hours spent gambling per day, the number of different active days gambled on, and the number of registered credit cards on the account were all higher compared to other clusters. Other criteria (e.g., removal of RG tools, cancelled withdrawals, abusive communication with customer services, third-party calls, and frequency of bonus begging) were lower than the mean for the total gamblers in the present sample (Fig. 2).

## Cluster 3—Emotionally Vulnerable Gamblers

**Fig. 3:**
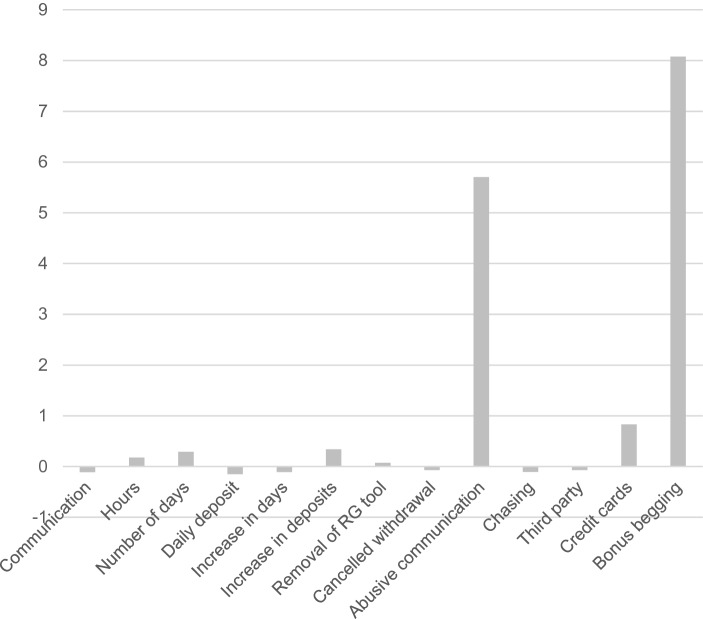
Behavioural metrics of the emotionally vulnerable cluster

The emotionally vulnerable cluster comprised nine gamblers (0.92%) and consisted of gamblers who scored most highly in relation to abusive communication with customer service staff and frequency of bonus begging. This appears to be a group of gamblers that experienced more emotional (rather than financial) problems. Out of the other criteria, six of these were lower than mean values in the total sample (communication with customer services, amount of daily deposit, increase in days, cancelled withdrawals, chasing and third-party calls) and seven were above average (hours, number of days, increase in deposits, removal of RG tools, abusive communication with customer services, number of registered credit cards, and frequency of bonus begging) (Fig. 3).

## Cluster 4—At-Risk Gamblers

**Fig. 4:**
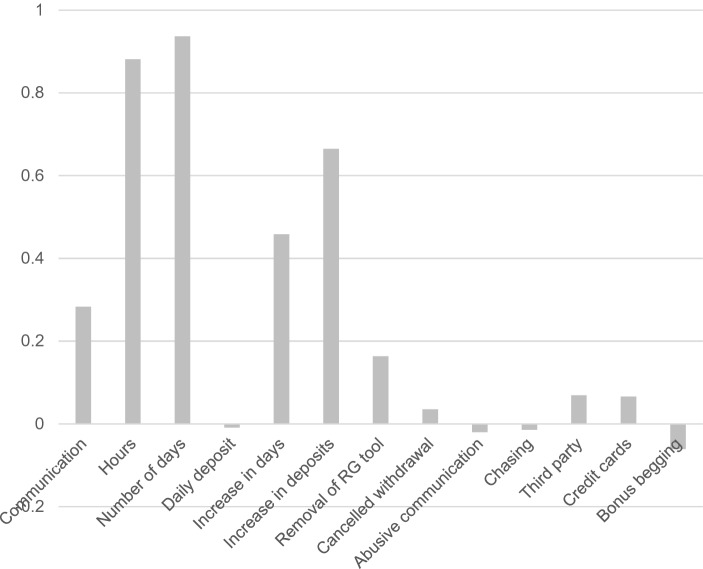
Behavioural metrics of the at-risk gambling cluster

The at-risk cluster comprised 324 gamblers (32.99%). This group had higher mean values than the rest of the sample on communication with customer services, hours spent gambling, number of active days gambling, increase in number of active days gambling over time, increase in number of deposits over time, removal of RG tools, cancelled withdrawals, third-party calls, and number of registered credit cards. Compared to the other clusters, four criteria had the highest value compared to all the other clusters in this cluster. These were communication with customer services, increase in deposits, removal of RG tools, and third-party calls (Fig. 4).

## Discussion

To the authors’ knowledge, the present study is the first to ever examine the application of the DSM-5 criteria of gambling disorder (APA, 2013) to actual gambling behaviour using online gambling transaction data. Initially (in the Introduction), each of the nine DSM-5 criteria were examined to see how these could be operationalized using account-based tracking data. This was done by consulting with experts from the online gambling industry, regulatory bodies, reformed problem gamblers, and other gambling researchers., as well as utilizing examples from the gambling studies literature After the DSM-5 criteria had been operationalized, the gambling behavioural indicators were analysed utilizing a sample of gamblers who had registered with the gambling operator *Unibet* from September 1 to December 31, 2018. For each gambler, the gambling activity in their first three months was analysed and operationalized in relation to the DSM-5 criteria for gambling disorder.

Initially, all the operationalized gambling disorder criteria were observed in the sample and the data were presented descriptively. In the second stage, the data were z-normalised to allow comparability and a two-step cluster analysis was performed. Four clusters emerged: non-problem gamblers (646 participants; 65.78%), financially vulnerable gamblers (three participants; 0.31%), emotionally vulnerable gamblers (nine participants; 0.92%), and at-risk gamblers (324 participants; 32.99%). In the first cluster (non-problem gamblers), all the gamblers had a negative z-score for all the proposed gambling disorder criteria. This showed that the majority of the population had values that were smaller than the means when compared to the other clusters.

The second cluster (financially vulnerable gamblers) comprised only three gamblers. In this cluster, it was evident that the number of daily deposits and the increase in daily deposits over time (i.e., chasing behaviour) were at much higher than compared to the mean values of the other clusters. The number of hours spent gambling online, the number of different active days gambled on, and the number of registered credit cards on the account were also higher than the mean values of the other clusters, but not as high as the number of daily deposits and the increase in daily deposits over time. On the other criteria, they were all close to the other cluster mean values, and in some measures (removal of RG tools, cancelled withdrawals, abusive communication, third-party calls, and frequency of bonus begging) were lower than the mean.

The third cluster (emotionally vulnerable gamblers) comprised nine gamblers. In this cluster, the most distinctive behavioural attributes were abusive communication with customer services staff and frequency of bonus begging. Six of the other criteria were negative z-scores, showing that the values were lower than the mean for the other clusters, whereas five of the criteria showed positive z-scores.

The final cluster (at-risk gamblers) comprised one-third of the gamblers. This cluster had no distinctive criteria, and all the values ranged from + 1 to − 1, and four of the criteria (number of daily deposits, abusive communication with customer services, chasing losses, and frequency of bonus begging) had negative z-scores, therefore the values were much lower to the total sample’s means. Frequency of bonus begging, abusive communication with customer services, number of daily deposits, and the increase in daily deposits over time were all lower than the mean of the other clusters. The other criteria had a positive z-score, with the number of active gambling days having the highest value of 0.937, showing that the z-score was almost one standard deviation greater than the mean of the total sample.

The benefits in the approach taken in the present study is that the whole population was observed and not just gamblers that chose voluntary self-exclusion (VSE) which has been the case in previous studies (e.g., Braverman & Shaffer, 2012; Haefeli et al., 2011; Percy et al., 2016). Griffiths and Auer (2016) highlighted that there are limitations in this latter approach because gamblers may not be using self-exclusion for problem gambling reasons. A recent study by Catania and Griffiths (2021) analysed 7732 gamblers who had used VSE. They reported almost one-fifth of the gamblers used the VSE option even though the players had less than 24 h of activity on their account. Moreover, gamblers who use VSE are treated by most researchers as a homogenous group despite the several differences present.

The majority of the sample in the present study were either in the non-problem gambling cluster (65.78%) or the at-risk gambling cluster (32.99%) comprising 98.77% the participants. In both groups, there were no distinctive criteria that were much higher than the means of the total sample which would be expected because the two clusters comprised a high percentage of the total population studied. This may show that these gamblers were playing within their financial means and not problematically. The remaining two clusters that did display elevated values on DSM-5 criteria for gambling disorder comprised 1.23% of the total sample. In the UK, the most recent British Gambling Prevalence Study reported that 0.9% of the population were problem gamblers using the DSM-IV criteria (Wardle et al., 2011). Therefore, the two clusters which comprise 1.23% of the total sample in the present study may reflect the individuals who are problem gamblers given the similarities in prevalence.

The financially vulnerable gambler cluster showed higher than average means in the number of daily deposits, increase in daily deposits over time (i.e., chasing behaviour), number of hours spent gambling, number of different active days spent gambling, and number of registered credit cards on the account. This group appeared to show much greater levels of preoccupying gambling behaviour based on the amount of time and money spent (Griffiths, 2012). Overall engagement and increase in time spent gambling are key predictors of gambling harm (Gainsbury et al., 2019). A high amount of deposited money has also been associated with gamblers that self-excluded for problem gambling reasons (Ukhov et al., 2021). The gamblers that were in this cluster also showed more indicators of chasing behaviour compared to the other clusters, and this may result in relying on others to provide money to relieve desperate financial situations that were caused by gambling (bailout). Gamblers may use bailout money by depositing it on their online gambling account, in order to chase their losses. In the present study, the number of registered credit cards on the gambling account may be an indicator for bailout, and was also higher compared to the mean of the total sample.

The psychologically vulnerable gambler cluster included two behaviours which were higher than the means of the total sample. These were abusive communication with customer services staff and frequency of bonus begging. These two behaviours may be signs of withdrawal and bailout. Gamblers may use aggressive communication in online gambling chatrooms (Griffiths, 2012), and gamblers may use abusive communication due to psychological and/or emotional strain that occurs due to problem gambling (Haefeli et al., 2011). This strain may be due to the losses being out of control for the gambler, or financial funds running out and therefore not being able to continue gambling. Bonus begging may be used in order for the gambler to get ‘free money’ on their account to be able to chase their losses and use this ‘free money’ as means to gamble when the financial means to do so are running low. The findings presented here may help gambling operators in minimising harm caused by gambling, because online behavioural tracking identify potentially problematic gambling behaviour (Haeusler, 2016). Additionally, using objective data collected by tracking technology, may overcome the weaknesses that are present with self-report data such as lying or the social desirability effect (Griffiths, 2009).

### Limitations

Although the present study may help in the discussion of operationalizing online behavioural tracking data and potential gambling disorder indicators, it does have a number of limitations. The dataset is from only one gambling operator, and this may be limited view since most gamblers use more than one gambling operator, and therefore generalisation to all online gamblers may be difficult to conclude (Auer & Griffiths, 2019; Auer et al., 2020). Some of the indicators developed are arguably quite novel (e.g., frequency of bonus begging) and therefore further research should be performed in relation to their reliability as indicators of potential gambling harm indicators. Another limitation could be that some of the operational definitions of each DSM-5 criterion were arguably narrow. For instance, in the present study chasing was simply defined as an increase in deposits over time. Further research should look at longitudinal aspects to see how these potential problem gambling indicators develop over time. Another approach could be to use self-report data in combination with these potential problem gambling indicators to better understand their prevalence with gambling-related harm.

### Conclusions

Despite its limitations, the present study creates a foundation of potential new problem gambling indicators that may be used in conjunction with the collecting of online behavioural tracking data. Furthermore, through analysing a whole gambling population and not limiting it to voluntary self-exclusion as a proxy measure for problem gambling, can further help gambling operators to prevent disordered gambling.

## Data Availability

Anonymised data provided by *Kindred Group.*
